# CD4 Count Pattern and Demographic Distribution of Treatment-Naïve HIV Patients in Lagos, Nigeria

**DOI:** 10.1155/2012/352753

**Published:** 2012-09-26

**Authors:** Akinsegun Akinbami, Adedoyin Dosunmu, Adewumi Adediran, Sarah Ajibola, Olajumoke Oshinaike, Kikelomo Wright, Olanrewaju Arogundade

**Affiliations:** ^1^Department of Haematology and Blood Transfusion, College of Medicine, Lagos State University, Ikeja, PMB 21266, Lagos, Nigeria; ^2^Department of Haematology and Blood Transfusion, Faculty of Clinical Sciences, College of Medicine, University of Lagos, PMB 12003, Idiaraba, Lagos, Nigeria; ^3^Department of Haematology and Blood Transfusion, Lagos University Teaching Hospital, PMB 12003, Idiaraba, Lagos, Nigeria; ^4^Department of Medicine, College of Medicine, Lagos State University, Ikeja, PMB 1266, Lagos, Nigeria; ^5^Department of Community Health and Primary Health Care, College of Medicine, Lagos State University, Ikeja, PMB 21266, Lagos, Nigeria

## Abstract

*Background*. CD4 count measures the degree of immunosuppression in HIV-positive patients. It is also used in deciding when to commence therapy, in staging the disease, and in determining treatment failure. Using the CD4 count, this study aimed at determining the percentage of HIV-positives who require antiretroviral therapy at enrollment in an HIV treatment and care centre. *Methods*. The Baseline CD4 count, age and gender of 4,042 HAART-naïve patients, who registered between December 2006 and June 2010, at Lagos State University Teaching Hospital, Ikeja, were retrospectively studied. Data were analyzed using SPSS version 16.0 (Statistical Package for Social Sciences, Inc., Chicago, Ill). *Results*. Patients consisted of 2507 (62%) female and 1535 (38%) males. The mean age of males was 37.73 ± 9.48 years and that of females 35.01 ± 9.34 years. Overall, the mean CD4 count was of 298.76 ± 246.93 cells/mm^3^. The mean CD4 count of males was 268.05 ± 230.44 cells/mm^3^ and that of females 317.55 ± 254.72 cells/mm^3^. A total of 72.3% males, 64.3% females and 67.4% overall registered patients had CD4 count <350 cells/mm^3^, while only 15.1% males , 20.3% females, and 18.3% overall registered patients had CD4 count >500 cells/mm^3^ at registration. *Conclusion*. Females account for more than half of registered patients in HIV clinic and have a relatively higher CD4 count than males. About three-quarter of HIV positives require antiretroviral therapy at registration.

## 1. Introduction

Worldwide Nigeria has the second highest number of new HIV/AIDS infections reported each year [1]. About 300,000 new infections occur annually with people aged 15–24 years contributing 60% of the infections and 1.5 million people living with HIV require antiretroviral using the new WHO guidelines. Only about 30% of people living with HIV who need antiretroviral have access to it [2].

In 2009 an estimated 3.6% of 150 million Nigerians are living with HIV and AIDS [3]. Approximately 215,000 people died of HIV/AIDS in Nigeria in 2010 [4].

CD4 count measures the degree of immunosuppression in HIV-positive patients. There is an inverse relationship between CD4 count and degree of immunosuppression. CD4 count is used in monitoring disease progression, deciding when to commence therapy, staging the disease, determining treatment failure, and defining the risk for mother-to-child transmission. 

Laboratory markers used in monitoring management in HIV-positive patients are HIV-RNA assay (Viral load) and CD4 count. The former is the gold standard, its use is, however, limited because of its cost and technology. Furthermore, there is a mismatch between an undetectable viral load (<50 copies/mL) and the absence of immune reconstitution, which can be confusing to both the treatment provider and patient.

Several studies have shown that CD4 count is the strongest predictor of disease progression and survival [5, 6]. The cost of CD4 count is cheaper than viral load, it is increasingly becoming more affordable to patients in resource-poor countries [7, 8]. All HIV-positive patients in resource rich and an increasing number of patients in resource-poor countries have baseline CD4 count on enrollment [9]. 

The CD4 cell count is the strongest predictor for risk of death and AIDS [10] at the time of initiating therapy, initiating highly active antiretroviral therapy (HAART) at higher CD4 cell counts has been demonstrated to “the risk of death, opportunistic infections and non-HIV related comorbidities” [11, 12]. Robert et al. [13] assessed CD4 count and the risk of death in HIV-infected patients on HAART, nearly all deaths occurred in patients with fewer than 50 CD4 cells/mm^3^.

A common denominator amongst all the guidelines on initiating HAART in HIV-positive patients is the use of CD4 count in deciding when to initiate ART in HIV-positive patients. While some HIV-positive patients present at registration with low CD4 count, that is, less than 200 cells/mm^3^ probably due to late presentation or diagnosis and are commenced on HAART almost immediately on enrollment irrespective of clinical symptoms. Some asymptomatic HIV-positive patients do not require antiretroviral (ART) drugs on enrollment because of their high CD4 count at registration hence their inability to meet criteria for initiation of therapy laid down by various organizations like World Health Organization (WHO), Centre for Disease Control, (CDC) Atlanta, Presidential Emergency Program for AIDS relief (PEPFER), Working Group of AIDS Research Advisory Council (OARAC), and European AIDS Clinical Society Guidelines, among others.

What is controversial is the optimal time to initiate antiretroviral therapy (ART). Various guidelines exist on the optimal time to initiate ART in adult asymptomatic patients. There seems to be consensus of opinion on deferral of ART in asymptomatic HIV patients whose CD4 count is greater than 500 cells/mm^3^. However, the revised 2010, the World Health Organization (WHO) [14] recommended that all adult and adolescent including pregnant women with HIV infection presenting with CD4 count ≤350 cells/mm^3^, should start ART regardless of the presence and absence of clinical symptoms. Those with severe and advanced clinical disease (WHO clinical stage 3 and 4) should start ART irrespective of their CD4 cell count.

The U.S. Department of Health and Human Services (DHHS) [15] recommended that treatment of HIV infection shold be commenced, in patients with CD4 counts between 350 and 500 cells/mm^3^. A randomized trial still in progress (START) [16] is randomizing people with a CD4 cell count of greater than 500 per *μ*L to either start antiretroviral therapy (ART) immediately or defer to a CD4 cell count of 350 per *μ*L. However, Severe et al. [17] recommended that access to antiretroviral therapy should be expanded to include all HIV-infected adults who have CD4+ T-cell counts of less than 350 cells/mm^3^ in those who live in areas with limited resources. 

The U.S. Centre for Disease Control (CDC) and the prevention [18] staging system used the CD4 count as a tool to stage HIV into categories A, B, and C based on whether the CD4 count is >500 cells/mm^3^, between 200–499 cells/mm^3^ and <200 cells/mm^3^, respectively. It defines AIDS as all HIV-positive patients with CD4 count <200 cells/mm^3^ or CD4% < 14%. On the contrary, the WHO staging is based on clinical findings and does not require CD4 count in order to accommodate for resource-constrained settings where CD4 count testing may not be available.

CD4 count is an important tool in determining treatment failure in HIV-positive patients. The 2010 World Health Organization (WHO) [14] revised guideline defined immunological failure as a fall of CD4 count to baseline level or below, or 50% fall from on-treatment peak value or persistent CD4 count below 100 cells/mm^3^. There must, however, be absence of concomitant infection to cause transient CD4 count decrease. A patient presenting with immunological or clinical failure (new or recurrent stage 4 disease) with viral load copies >5000 copies/mL is deemed to have treatment failure and switched to second-line regimens [14].

The introduction of HAART as a modality of treatment in HIV positives has resulted in a dramatic decrease in AIDS-related morbidity and mortality and a great improvement in CD4 count of patients [19]. In order to determine the true picture of CD4 count pattern in HIV positives, HAART-experienced patients must, therefore, be excluded from the study. There is paucity of data on the pattern of CD4 count of HIV-positive, HAART-naïve patients at registration in Nigeria. The data may be used to determine the percentage of HIV-infected patients who require ART at registration. This will assist clinicians and policy makers in determining the point to begin treatment and the percentage of infected patients who require treatment at registration. Thus, this study aimed at determining the percentage of HIV positives who require treatment at enrollment using the CD4 count as a tool.

## 2. Materials and Methods

The records of 4,042 HAART-naïve, HIV-positive patients who registered at the HIV clinic of Lagos State University Teaching Hospital (LASUTH), Ikeja, between December 2006 and June 2010 were retrospectively reviewed. 

Lagos is Nigeria most prosperous and arguably the most populous city. It has one of the highest standards of living as compared to other cities in Nigeria as well as Africa. All ethnic groups in Nigeria are well represented in Lagos because of its cosmopolitan constitution. LASUTH, the only teaching hospital owned by the State Government and one of the two in Lagos, is located in the state capital Ikeja. It serves as a referral centre to 26 other secondary (General) hospitals serving an estimated 17 million Lagosians. 

Data retrieved included baseline CD4 count, age, and gender. All HAART-experienced, registered patients referred from other centers were excluded from the study.

## 3. Statistical Analysis

Data were analyzed using SPSS version 16.0 (Statistical Package for Social Sciences, Inc., Chicago, Ill); a statistical computer software. The descriptive data were given as means ± standard deviation (SD). The differences were considered to be statistically significant when the *P* value obtained is less than 0.05.

## 4. Results

Data from 4,042 registered patients were reviewed, consisting of 2507 (62%) females and 1535 (38%) males (Table 1). The overall minimum age was 15years and the maximum 85 years with a mean of 36.04 ± 9.49 years (Table 1). A majority 2414 of 4042 (59.7%) of all patients were between 31–50 years, 1318 of 4042 (32.6%) between 15–30 years and only 310 of 4042 (7.7%) were older than 50 years (Table 1).

The minimum age for male was 15 years and the maximum of 85 years with a mean of 37.73 ± 9.48 years (Table 1). A majority of males, 1035 of 1535 (67.4%) were between 31–50 years, 364 of 1535 (23.7%) between 15–30 years and 136 (8.9%) older than 50 years (Table 1).

The minimum age for female was 15 years and the maximum 76 years with a mean of 35.01 ± 9.34 years (Table 2). The majority of females, 1379 of 2507 (55%) were between 31–50 years, 954 of 2507 (38.1%) between 15–30 years and 174 of 2507 (6.9%) older than 50 years (Table 1).

Overall, the minimum CD4 count was 2 cells/mm^3^ and the maximum 1868 cells/mm^3^ with a mean of 298.76 ± 246.93 cells/mm^3^ (Table 2). Seven hundred and forty-one of 4042 (18.3%) had CD4 count of >500 cells/mm^3^ consisting of 414 (55.8%) in the age group 31–50 years, 271 (36.57%) between 15–30 years and 56 (7.55%) older than 50 years. While 3301 of 4042 (81.7%) had CD4 count of <500 cells/mm^3^, consisting of 578 (14.3%) with CD4 count of between 350–500 cells/mm^3^, 2723 (67.4%) with CD4 <350 cells/mm^3^ and 1712 (42.4%) with CD4 count <200 cells/mm^3^. Only 522 of 4042 (12.9%) had CD4 count of <50 cells/mm^3^. This consists of 319 of 522 (61.1%) in the age group 31–50 years, 166 (31.8%) between 15–30 years, and 37 (7%) older than 50 years (Table 2). 

The minimum CD4 count for males was 3 cells/mm^3^ and the maximum 1416 cells/mm^3^ with a mean of 268.05 ± 230.44 cells/mm^3^ (Table 2). A total of 233 (15.1%) of 1535 male patients had CD4 count >500 cells/mm^3^ consisting of 169 of 233 (65.23%) between the age of 31–50 years, 60 (25.75%) between 15–30 years, and 21 (9%) older than 50 years. The majority 1302 of 1535 (84.9%) had CD4 count <500 cells/mm^3^. A total of 193 of 1535 (12.6%) had CD4 count between 350–500 cells/mm^3^, while 1109 of 1535 (72.3%) had CD4 count <350 cells/mm^3^, 740 of 1535 (48.3%) with CD4 count <200 cells/mm^3^. Only 245 (16%) of males had CD4 count <50 cells/mm^3^ consisting of 169 (68.97%) between 31–50 years, 51(20.81%) between 15–30 years, and 25 (10.2%) older than 50 years (Table 2); *P* = 0.528.

The minimum CD4 count for females was 2 cells/mm^3^ and the maximum of 1868 cells/mm^3^ with a mean of 317.55 ± 254.72 cells/mm^3^ (Table 2). A total of 508 of 2507 (20.3%) had CD4 count >500 cells/mm^3^ consisting of 262 of 508 (51.57%) between 31–50 years, 211 of 508 (41.53%) between 15–30 years and 35 (6.8%) older than 50 years. A total of 1999 of 2507 (79.7%) females who had CD4 count <500 cells/mm^3^ consisted of 385 of 2507 (15.4%) with CD4 count of between 350–500 cells/mm^3^ 1614 of 2507 (64.3%) of females with CD4 count <350 cells/mm^3^, and 972 of 2507 (38.77%) with CD4 count <200 cells/mm^3^. A total of 277 of 2507 (11%) had CD4 count <50 cells/mm^3^ consisting of 150 of 277 (54.15%) between 31–50 years, 115 of 277 (41.51%) between 15–30 years, and 12 of 277 (4.3%) older than 50 years (Table 2); *P* = 0.039.

## 5. Discussion

In Nigeria, the first case of HIV/AIDS was reported in 1986. HIV prevalence declined from 6% in 2001 to 4.3% in 2005, 4.2% in 2008, and 4.1% in 2010. HIV response in Nigeria was health sector driven from 1986–1989,, but a multisectoral response commenced in 2000. Funding for the HIV response in Nigeria is obtained from both domestic (Federal Government of Nigeria, private sectors and state governments) and international sources like the U.S. Government, DFID, UN agencies, and global funds. It is pertinent to determinethe percentage of HIV-infected patients who require ART at registration vis-à-vis percentage benefiting from care services in order to appreciate progress made in reaching out to those in need of accessing care and treatment.

In Nigeria, prevalence among young women aged 15–24 years is estimated to be three times higher than among men of the same age. Females constitute 58% (about 1.72 million) of persons living with HIV in Nigeria and each year, 55% of AIDS death occurs among women and girls [2] The female:male ratio in the present study of 1.6 : 1 is similar to 1.8 : 1 obtained by Omoti et al. [20] in Benin City, Nigeria. The disparity in gender prevalence is age dependent as reported by Glynn et al. [21] who reported HIV prevalence was six times higher in women than in men amongst sexually active 15–19 years old, but it drops to three times that in men among 20–24 years old and equal to that of men among 25–49 years old. The present study did not consider age in groups in relation to gender and HIV status. However, 59.7% of the studied population was between 31–50 years, a ratio of F : M of 1.6 : 1 obtained was, therefore, similar to 1 : 1 reported by Glynn et al. in those between 25–49 years. Generally, females are more predisposed to contracting HIV because of pregnancy or use of oral contraceptive, conditions which induced cervical ectopia in which there is replacement of squamous by columnar epithelium, thus increasing the risk of HIV infection for women 5-fold. Sexual intercourse during menstruation and presence of genital ulcer also increases the risk of HIV infection in females. Pelvic inflammatory disease predisposes to microulceration of the genital tract thus increasing risk of HIV infection. Culturally, the majority of males in this part of the world are circumcised, male circumcision affords some degree of protection, perhaps due to large numbers of langerhans cells in foreskin, so that the incidence of infection is reduced 8-fold over uncircumcised men [22]. Glynn reported that, despite all the predisposing factors in females, women married younger than men, and marriage was a risk factor for HIV, women often had older partners and men rarely had partners much older than themselves.

The mean ages of 35.01 ± 9.34 years and 37.73 ± 9.48 years for females and males, respectively, obtained in this study are similar to earlier study [21] of 34.41 ± 8.87 and 38 ± 9.35 for females and males, respectively, and also similar to 38 years reported by Omoti et al. [20] in both genders. This is understandably so because the majority of patients in HIV clinic are between 31–50 years being the age when sexual activity is at its peak.

This study reported mean CD4 counts in HAART-naïve, HIV positives of 268.05 ± 230.44 and 317.55 ± 254.72 cells/mm^3^, respectively, for males and females and an overall mean of 298.76 ± 246.93 cells/mm^3^. This could be compared with 303.16 ± 234.32 cells/mm^3^ and 308.24 ± 232.2 cells/mm^3^, respectively, for males and females and an overall mean CD4 count of 306.65 ± 232.24 cells/mm^3^ reported by Akinbami et al. in an earlier study [23]. In both studies, females were found to a have a higher CD4 count than males.

Oladepo et al. [24] established in healthy Nigerian adults a reference value for CD4 of 365 to 1,571 cells/*μ*L. with a mean CD4 count of 847 cells/*μ*L similar to the mean value of 828 cells/*μ*L reported by Aina et al. [25] in an earlier study in Nigeria. Females were found to have significantly higher values of absolute CD4 counts in Oladepo's study in contrast to the earlier limited study by Aina et al. in Nigeria. This observation of higher CD4 count in females was also reported in several other countries among Nigerians [26], Ugandans [27], and Ethiopians [28]. A sex hormone effect is one possible explanation for the reported difference in CD4 counts between genders that has been suggested [28]. 

Using the 350 cells/mm^3^ CD4 count as cutoff from 2010 WHO [14] revised guideline for initiation of therapy in asymptomatic HIV patients, 72.3% males, 64.3% females and 67.4% overall registered patients require treatment on enrollment, while only 15.1% males, 20.3% females, and 18.3% overall registered patients with CD4 count >500 cells/mm^3^ may require antiretroviral therapy deferral at registration.

In Sub-Saharan Africa, an estimated 10 million need treatment in 2010, only 5 million received it [1]. In Nigeria as at 2009, only 31% of people living with HIV have access to care services [29], the government, through the National HIV/AIDS strategic framework for 2005–2009, set out to provide ARV to 80% of adults and children with advanced HIV infection and 80% of HIV-positive pregnant women, all by 2010 [30].

ARV treatment coverage in Nigeria remains low, the slow progress led to revising the strategic framework, and resetting treatment goals in its revised 2010–2015 framework [31]. By 2010, only a quarter of adults and 7% of children in need of treatment received it. Currently, 1.4 million adults and 262,000 children eligible for antiretroviral treatment remain without it [1].

In Africa, Botswana at the end of 2010 has the highest coverage rate around 93%, other countries that have achieved more than 80% coverage are Rwanda and Namibia [1]. Access to antiretroviral therapy in Somalia is the lowest in Africa at 3% while only 55% of those in need in South Africa are receiving it [32].

Cameroon, Cote d'Ivoire, Chad, Nigeria, and Ghana are some of the countries in Sub-Saharan Africa where between 20–39% of people requiring antiretroviral drugs are receiving them [1]. 

Being a retrospective study, some of the limitations of this study is the nonavailability of data on clinical manifestations of the patients, lack of data on records of distribution of the CD4 count, and percentage in need of treatment per year and lack of information on HIV risk factor, area of residence, family income, and marital status.

Extra efforts must be made by the Nigerian Government at all levels to meet the need of people living with HIV/AIDS eligible for treatment so as to reduce the spread of the pandemic.

## 6. Conclusion

About three-quarter of HIV positives require antiretroviral therapy at registration when 2010 WHO criteria are used for initiation of therapy, female population in HIV clinic is higher than males and the former has a relatively higher CD4 counts than the latter.

## Figures and Tables

**Table 1:** Gender, age, and CD4 count frequency.

Gender/Age	Male	Female	Overall
Gender	1535 (4042)	2507 (4042)	4042
	38%	62%	100%
Age (years)			
15–30	364 (1535)	954 (2507)	1318 (4042)
	23.7%	38.1%	32.6%
			
31–50	1035 (1535)	1379 (2507)	2414 (4042)
	67.4%	55%	59.7%
			
>50	136 (1535)	174 (2507)	310 (4042)
	8.9%	6.9%	7.7%
			
Mean age	37.73 ± 9.48	35.01 ± 9.34	36.04 ± 9.49
			
CD4 count cells/mm^3^	268.05 ± 230.44	317.55 ± 254.72	298.76 ± 93

**Table 2:** CD4 counts distribution according to age and gender categories.

Age categories (Years)	Male	Female	Overall
CD4 counts cells/mm^3^			
	>500: 233 (1535) 15.2%	508 (2507) 20.3%	741 (4042) 18.3%
15–30	60 (233) 25.8%	211 (508) 41.5%	271 (741) 36.6%
31–50	169 (233) 72.5%	262 (508) 51.6%	431 (741) 58.1%
>50	21 (233) 9%	35 (508) 6.9%	53 (741) 7.2%
	<500: 1302 (1535) 84.8%	1999 (2507) 79.7%	3301 (4042) 81.7%

CD4 count ranges incells/mm^3^			
350–500	193 (1535) 12.6%	385 (2507) 15.4%	578 (3301) 17.5%
<350	1109 (1535) 72.2%	1614 (2507) 64.4%	2723 (3301) 82.5%
<200	740 (1535) 48.2%	972 (2507) 38.8%	1712 (3301) 51.9%
<50	245 (1535) 16%	277 (2507) 11%	522 (3301) 15.8%
